# Investigation of the Possible Causes of NRBC in ICU Patients and the Dynamic Trend of NRBC Count in Survival and Death Patients or with Different Underlying Diseases: A Retrospective Study

**DOI:** 10.1155/2023/9076647

**Published:** 2023-01-14

**Authors:** Jing Wang, Zhengxian Su, Xinzhuan Zhao, Xiaxia Jin, Guoguang Lu, Yuan Yuan, Tao Li

**Affiliations:** ^1^Department of Clinical Laboratory, Taizhou Hospital of Zhejiang Province Affiliated to Wenzhou Medical University, 150 Ximen Road, Linhai, Taizhou, Zhejiang, China; ^2^Department of Cardiovascular Medicine, Taizhou Hospital of Zhejiang Province Affiliated to Wenzhou Medical University, 150 Ximen Road, Linhai, Taizhou, Zhejiang, China

## Abstract

**Background:**

The mortality of intensive care unit (ICU) patients ranges from 5% to 30%, and nucleated red blood cells (NRBCs) were revealed to be related to mortality. However, few studies have discussed the causes of NRBC or compared the dynamic count among patients with underlying diseases.

**Aim:**

To explore the possible causes of NRBC in ICU patients and the dynamic trends between survival and death groups and underlying disease subgroups.

**Methods:**

A total of 177 ICU patients were retrospectively included. The possible causes of NRBC in ICU patients were discussed. The relationship between NRBC and in-hospital mortality and the dynamic trend of NRBC during hospitalization between the survival and death groups and underlying disease subgroups were compared.

**Results:**

The Acute Physiology and Chronic Health Evaluation II (APACHE II) score and Sequential Organ Failure Assessment (SOFA) score in the NRBC-positive group were higher (23.52 ± 9.39 vs. 19.62 ± 7.59; 13.50 (9.00–17.50) vs. 8.00 (6.00–12.00)). Red blood cell count (RBC), hemoglobin (Hb) level, oxygen saturation (SO_2_), oxygenation index (OI), and serum protein level were lower in the NRBC-positive group. However, D-dimer (D-D), liver and kidney function indices, lactate dehydrogenase (LDH), C-reactive protein (CRP), and procalcitonin (PCT) were higher than those in the NRBC-negative group. Correlation analysis showed that NRBC count was positively correlated with alkaline phosphatase (ALP) and red blood cell distribution width (RDW) and negatively correlated with SO_2_ (*r* = 0.431, *P* < 0.05; *r* = 0.363, *P* < 0.05; *r* = −0.335, *P* < 0.05). The mortality rate in the NRBC-positive group was higher, and the median survival time was shorter than that in the NRBC-negative group (77.9% vs. 95.7%, *P* < 0.001; 15 days vs. 8.5 days, *P* < 0.01). Univariate and multivariate Cox regression analyses showed that NRBC was an independent risk factor for in-hospital mortality (HR: 1.12 (1.03–1.22), *P* < 0.01). The NRBC count had different hazard ratios (HRs) for in-hospital mortality in the subgroups. Locally weighted scatterplot smoothing (LOWESS) analysis revealed that the NRBC count in the death group was higher and had a sharp upward trend before death, whereas that in the survival group was negative or stayed at a low level. The changing trend of the NRBC count was different in patients with different underlying diseases.

**Conclusion:**

The possible cause of NRBC in ICU patients was related to inflammation and hypoxia. The persistently high level and rapid upward trend of NRBC counts are risk factors for in-hospital mortality in ICU patients. The changing trend of the NRBC count varied in patients with different underlying diseases.

## 1. Introduction

Mortality rates ranged from 5% to 30% in critically ill patients, who are considered case sensitive [1, 2]. Multiple factors, including the treatment condition and severity degree, are related to mortality. The mortality prediction of critically ill patients is challenging but can be helpful for general counseling, triaging, treatment decisions, and end-of-life discussions [3].

Previous studies have indicated that nucleated red blood cells (NRBCs) are related to the mortality of patients from the intensive care unit (ICU), and the indices of NRBC used for prediction include the NRBC-positive rate [4, 5] and NRBC counts [6]. However, most studies were limited to comparing the mortality of NRBC-negative and NRBC-positive patients or the NRBC counts between the survival and death groups.

However, the possible causes of NRBC, subgroup analysis for the risk of NRBC for in-hospital mortality, and dynamic change trends in survival and death groups or patients with different underlying diseases have not been discussed in detail. In this study, we recorded the underlying conditions, clinical scores, and laboratory results on the admission of all patients, and we also conducted 106 days of dynamic tracking on the NRBC count of peripheral blood during hospitalization to explore the possible causes of NRBC in ICU patients and the correlation between NRBC and in-hospital mortality, and we further discussed the dynamic change trend of NRBC in subgroups of patients.

## 2. Materials and Methods

### 2.1. Sample Size and Power Estimation

G^*∗*^ Power (version 3.1.9.7) was used for sample size and power estimation. The parameter settings are as follows: effect size *d* = 0.5, *α* = 0.05, 1 − *β* = 0.8, allocation ratio N2/N1 = 0.4. After calculation, the total number of patients required for the study was 164 cases, including 117 cases in the NRBC-negative group and 47 cases in the NRBC-positive group. The actual power was 0.803.

### 2.2. Patient Selection and Data Collection

Two hundred twenty-four inpatients in the ICU from August 1, 2018, to May 26, 2019, were retrospectively collected. Thirty-six patients with routine ICU care after extensive organ surgery and 11 patients without NRBC tests within three days after admission were excluded, and 177 were finally selected. The results of the complete blood count (CBC), blood biochemistry, coagulation function, inflammatory indices, and blood gas analysis were recorded within three days after admission. Furthermore, all the NRBCs counts during hospitalization were collected.

### 2.3. Laboratory Sample Processing and Detection

Approximately 10 ml of fasting venous blood samples were collected from ICU patients within three days of admission. Two milliliters were placed in a vacuum tube containing EDTA-K_2_ anticoagulant (BD Company, New Jersey, USA) and automatically detected in the assembly line of the Mindray BC-6800 plus automated hematology system (Mindray, Shenzhen, China) for the CBC indicators including white blood cell (WBC), neutrophils (N), red blood cell (RBC), hemoglobin (Hb), red blood cell distribution width (RDW), NRBC, and platelet count (PLT).

Approximately 2.7 ml of each sample was placed into a vacuum tube containing sodium citrate anticoagulant (BD Company, New Jersey, USA) and automatically detected for D-dimer (D-D) and fibrinogen (Fig) by Stago StaR Max (Stago, Paris, France).

The remaining venous blood was placed in two tubes with a coagulant (BD Company, New Jersey, USA) and centrifuged at 3500 rpm for 5 minutes for serum. The serum samples were tested by the Abbott C16000 automatic biochemical analyzer (Abbott Laboratories, Illinois, USA). The results included alanine aminotransferase (ALT), aspartate aminotransferase (AST), alkaline phosphatase (ALP), *γ*-glutamine transferase (GGT), total bilirubin (TBIL), direct bilirubin (DBIL), prealbumin (PA), total protein (TP), albumin (Alb), albumin/globulin (A/G), total bile acid (TBA), cholinesterase (CHE), crea (Cr), blood urea nitrogen (BUN), uric acid (UA), estimated glomerular filtration rate (eGFR), potassium (K), sodium (Na), chlorine (Cl), calcium (Ca), magnesium (Mg), phosphorus (P), c-reactive protein (CRP), serum amyloid A (SAA), high-density lipoprotein cholesterol (HDLC), low-density lipoprotein cholesterol (LDLC), apolipoprotein A1 (APOA1), apolipoprotein B (APOB), total cholesterol (TC), lactate dehydrogenase (LDH), and complement 3 (C3). Procalcitonin (PCT) was tested on a Cobas E601 automatic electrochemiluminescence analyzer (Roche, Basel, Switzerland).

At the same time, 2 ml of arterial blood was drawn and placed in a unique tube (Roche, Basel, Switzerland) for blood gas analysis and detection by a Cobas b121 automatic blood gas analyzer (Roche, Basel, Switzerland). The results, including the partial pressure of oxygen (PO_2_), partial pressure of carbon dioxide (PCO_2_), oxygen saturation (SO_2_), and oxygenation index (OI), were recorded. All samples were mixed immediately after extraction.

### 2.4. Clinical Scoring Systems

The Charlson comorbidity index (CCI) was used to assess the burden of chronic illness, which is well studied and validated. Charlson introduced the CCI in 1987. It refers to the injury and abnormality of other organs or tissues except for the underlying diseases. It performs a comprehensive evaluation of the patients' complications, such as congestive heart failure, myocardial infarction, cerebrovascular disease, dementia, peripheral vascular disease, chronic obstructive pulmonary disease, peptic ulcer disease, connective tissue disease, diabetes, moderate-severe chronic kidney disease, hemiplegia, leukemia, lymphoma, solid tumor, and liver disease [7].

APACHE II was utilized to evaluate the severity of ICU patients. The APACHE II score is the sum of the acute physiology score, age score, and chronic health score. The acute physiology score includes body temperature, heart rate, respiration, blood pressure, PO_2_, and pH, K, Na, Cr, WBC, and HCT, and the worst value of each index was recorded within 24 hours of entering the ICU [8].

We used the SOFA score to determine the number of failed organs. The SOFA score included respiratory, blood, circulatory, neurological, and renal indices. The worst value of the daily assessment was recorded [9].

### 2.5. Grouping of Enrolled Patients and Study Design

The NRBC count at admission was recorded as the highest NRBC count within three days of admission. According to the results, patients were divided into NRBC-positive and NRBC-negative groups.

**Figure 1:**
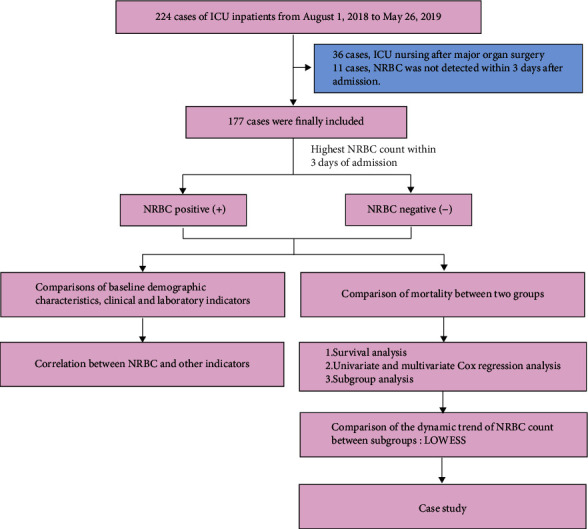
Flowchart of the study cohorts.

The possible causes of NRBC in ICU patients were discussed by the correlation between the NRBC count and other indicators. At the same time, the in-hospital mortality and the survival time of the NRBC-positive and NRBC-negative groups were compared. Moreover, the relationship between NRBC and in-hospital mortality of ICU patients was compared through survival analysis, univariate and multivariate Cox regression analyses, and subgroup analysis. Then, the dynamic change trend of the NRBC count in subgroups was compared. Finally, the dynamic changes in NRBC count in representative patients are shown (Figure 1).

### 2.6. Statistical Analysis

R (Version: 4.0.2, Vienna, Austria, https://www.R-project.org/) was used for all statistical analyses and all charts. Categorical variables were expressed in numbers (percentages), and a chi-square test was used for intergroup comparisons. The normality of the data was determined by a Shapiro–Wilk test. Continuous variables conforming to a normal distribution were expressed as the mean ± standard deviation, and comparisons between groups were conducted by a *t*-test; otherwise, they were expressed as the median (P25–P75), and a Mann–Whitney *U* test was used for intergroup comparisons. Spearman correlation analysis was used to analyze the correlation of the indicators. Risk factors were screened by univariate and multivariate Cox regression analyses. Subgroup analysis was used to observe the relationship between NRBC and in-hospital mortality of ICU patients in each subgroup. The dynamic change trend was represented by locally weighted scatterplot smoothing (LOWESS). *P* < 0.05 stands for statistical significance.

## 3. Results

### 3.1. Comparison of Demographic Characteristics and Clinical and Laboratory Indices between the NRBC-Positive Group and NRBC-Negative Group

**Figure 2:**
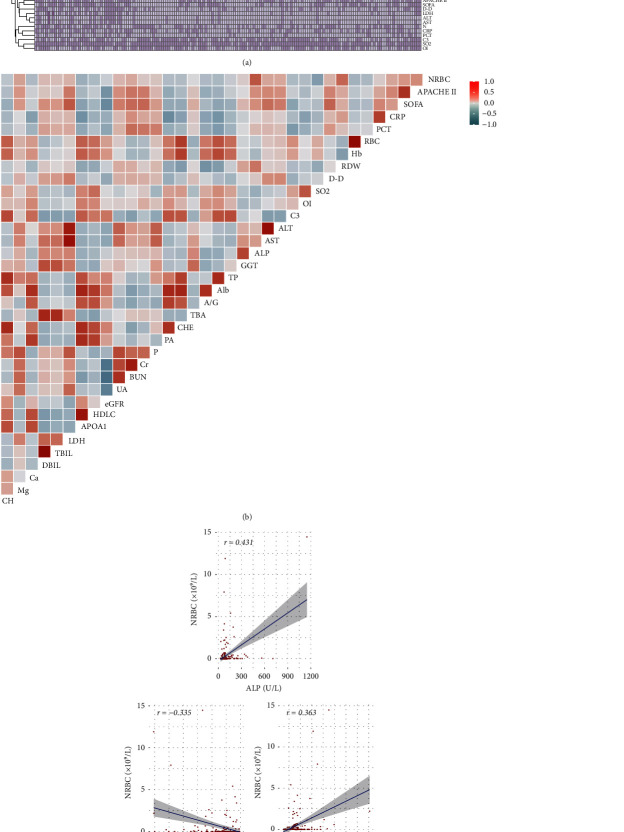
Heatmaps and correlations between NRBC and other indicators. (a) Heatmaps of clinical and laboratory indicators. (b) Correlations between NRBC and other indicators. (c) Correlations of NRBC and ALP, SO_2_, and RDW.

The results showed no significant difference in the sex ratio or age between the two groups. Among the underlying diseases, the proportion of urinary system diseases in the NRBC-positive group was higher than that in the NRBC-negative group, while the proportion of cardiovascular and cerebrovascular diseases and hypertension was lower (18.3% vs. 34.8%; 67.9% vs. 43.5%; 37.4% vs. 19.6%), but there was no significant difference in CCI score between the groups. The APACHE II score and SOFA score of the NRBC-positive group were higher (23.52 ± 9.39 vs. 19.62 ± 7.59; 13.50 (9.00–17.50) vs. 8.00 (6.00–12.00)). Regarding treatment, the NRBC-positive group had a higher proportion of blood transfusions, hemodialysis/continuous renal replacement therapy (CRRT), and extracorporeal membrane oxygenator (ECOMO). In the complete blood count, the number of RBC and Hb levels in the NRBC-positive group was lower, and RDW was higher than that in the NRBC-negative group. Among the coagulation function indices, the D-D level in the NRBC-positive group was higher. The oxygen saturation (SO_2_) and oxygenation index (OI) in the NRBC-positive group were lower than the blood gas indices. The indices of liver function (ALT, AST, ALP, GGT, TBA, TBIL, and DBIL), renal function (Cr, BUN, and UA), electrolyte (Mg, P), myocardial enzyme (LDH), and infection index (CRP and PCT) in the NRBC-positive group were higher than those in the NRBC-negative group, while serum protein (PA, TP, ALB, and A/G), blood lipids (TC, HDLC, and ApoA1), and complement 3 (C3) were lower (Table 1, Figure 2(a)).

### 3.2. Correlation Analysis between NRBC Count, Clinical Score, and Other Laboratory Indices in ICU Patients

The correlation between NRBC and other indicators is shown in Figure 2(b). The correlation coefficient between ALP, RDW, and SO_2_ and NRBC count was higher than 0.3, in which NRBC count was positively correlated with ALP and RDW and negatively correlated with SO_2_ (*r* = 0.431, *P* < 0.05; *r* = 0.363, *P* < 0.05; *r* = −0.335, *P* < 0.05). In addition, NRBC was also positively correlated with the APACHE II score, SOFA score, CRP, and PCT (Figure 2(c)).

### 3.3. Comparison of Mortality between NRBC-Positive and NRBC-Negative Groups and Risk Factors for In-Hospital Mortality in ICU Patients

**Figure 3:**
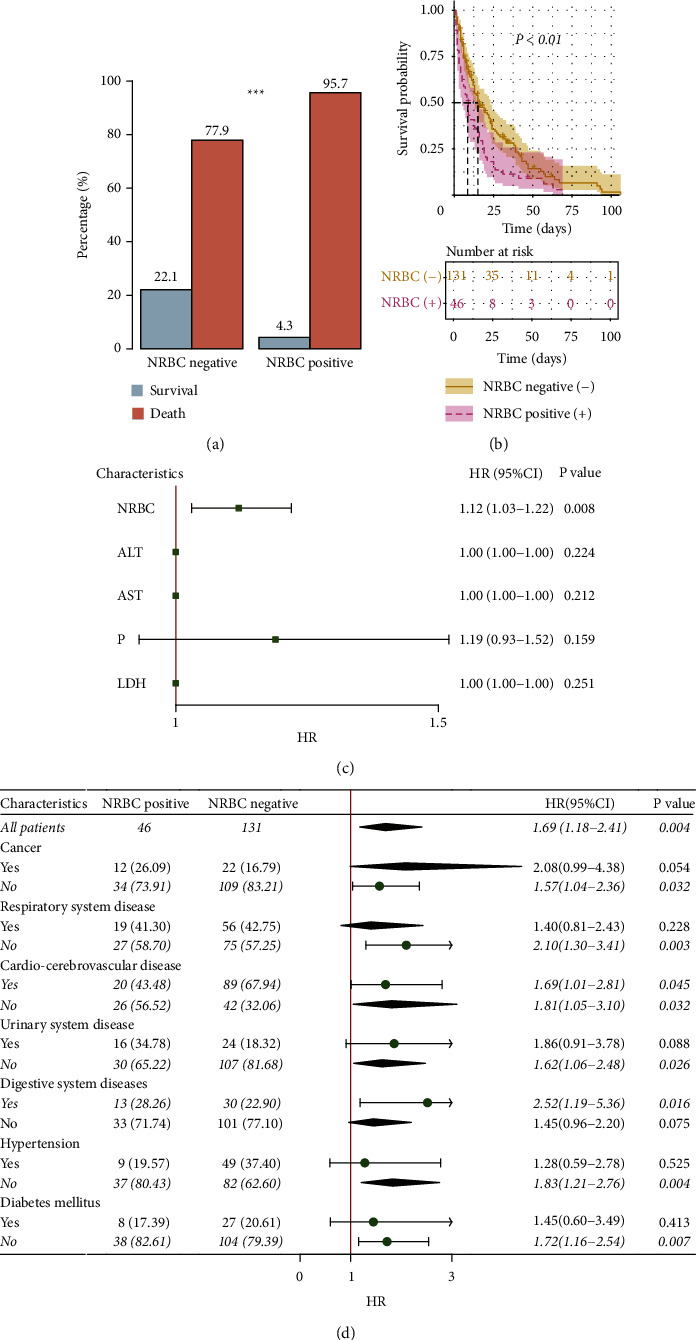
The effects of NRBC on mortality in ICU patients include survival analysis, multivariate cox regression analysis, and subgroup analysis. (a) Death and survival rates of NRBC-positive and NRBC-negative groups. (b) Survival analysis of NRBC-positive and NRBC-negative groups. (c) Forest plot of multivariate cox regression. (d) Subgroup analysis of different underlying diseases.

The mortality of patients in the NRBC-negative group was 77.9%, while that in the NRBC-positive group was 95.7% (Figure 3(a)). The survival curve showed that the median survival time of the NRBC-negative group was longer than that of the NRBC-positive group (15 days vs. 8.5 days, *P* < 0.01) (Figure 3(b)). The univariate Cox regression analysis included the clinical and laboratory indices, with significant differences between the groups. The results showed that ALT, AST, LDH, NRBC, P, Hb, APACHE II score, and TBIL might be risk factors. Then, the above indices were included in the multivariate Cox regression analysis, and NRBC was revealed to be an independent risk factor for in-hospital mortality in ICU patients (HR: 1.12 (1.03–1.22), *P* < 0.01) (Figure 3(c)). In addition, we further conducted a subgroup analysis. The results showed that the mortality risk of NRBC-positive patients was higher than that of NRBC-negative patients in the overall population (HR: 1.69 (1.18–2.41), *P* < 0.01). The subgroups of different underlying diseases showed that in patients without malignant tumors, respiratory diseases, cardiovascular and cerebrovascular diseases, urinary diseases, hypertension, and diabetes, the mortality risk was higher in the NRBC-positive group. The same was true for patients with digestive system disease (*P* < 0.05) (Figure 3(d)).

### 3.4. Comparison of NRBC Dynamic Change Trends between Surviving and Nonsurviving Patients in Each Subgroup

**Figure 4:**
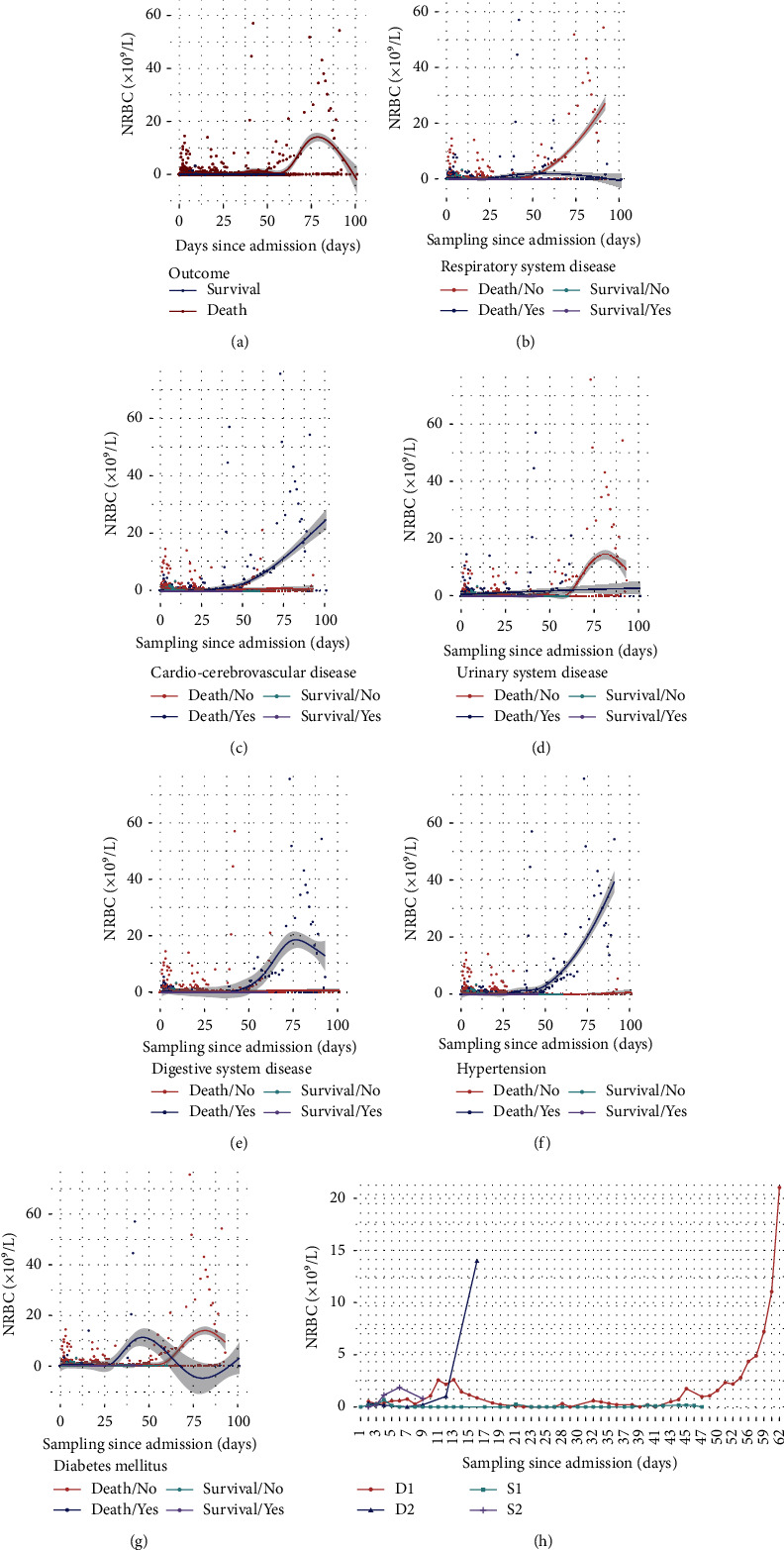
Comparisons of NRBC dynamic trend between subgroups and case analysis between NRBC-positive and NRBC-negative patients. (a) Survival and death groups. (b) With/without respiratory system disease. (c) With/without the cardio-cerebrovascular disease. (d) With/without urinary system disease. (e) With/without digestive system disease. (f) With/without hypertension. (g) With/without diabetes mellitus. (h) Representative case analysis.

The dynamic changes in NRBC count showed that the NRBC count in the nonsurviving group was higher than that in the surviving group during hospitalization, especially in the early stage. The subgroup analysis of dead patients showed that the NRBC count of patients with respiratory diseases was low, while the NRBC count of patients without respiratory diseases increased sharply from 50 days after admission to death. The NRBC count of patients without cardiovascular and cerebrovascular diseases was low, while the NRBC of patients with cardiovascular and cerebrovascular diseases increased sharply from 30 days after admission to death. The NRBC count of patients with urinary system diseases increased steadily from admission to death, while the NRBC count of patients without urinary system diseases increased sharply at 60 days after admission. Although it decreased by approximately 80 days, it remained at a high level. The patients without digestive system diseases were maintained at a nearly negative level throughout hospitalization, while the patients with digestive system diseases showed a strong upward trend from 40 days to 75 days after admission. Although they decreased after 75 days, they were still maintained at a high level. In patients with hypertension, NRBC rose sharply approximately 30 days after admission and reached the highest value of approximately 40 × 10^9^/L at the time of death. In addition, after 30 days of admission, the NRBC count of patients without diabetes began to increase and gradually decreased from 45 days of admission and tended to be negative before death. The number of patients without diabetes gradually increased approximately 60 days after admission and dropped slightly after approximately 80 days, but remained high (Figures 4(a)–4(g)).

### 3.5. Representative Case Analysis

We selected four representative cases in the NRBC-positive group, including two dead cases (D1, D2) and two surviving cases (S1, S2), and observed the quantitative results of the NRBC count during their survival period. The results showed that the NRBC count of dead patient D1 repeatedly increased during hospitalization and increased sharply between 42 days and 62 days after admission. D2 had a low NRBC count at admission (0.16 × 10^9^/L), which was then sustained at zero until it began to rise sharply one week before death and reached its highest value on the day of death (14 × 10^9^/L). The NRBC count of patient S1 was occasionally positive, with a low value during hospitalization. Although the NRBC count of the surviving patient S2 was positive during hospitalization, the values were within 2 × 10^9^/L (Figure 4(h)).

## 4. Discussion

In this study, we found that NRBC-positive patients had more underlying diseases and a higher probability of severe conditions. The cause of NRBC in peripheral blood was related to oxygen deficiency, low protein level, hypercoagulability, hyperinflammation, and impaired function of multiple organs. NRBC was an independent risk factor for mortality in ICU patients, and NRBC-positive patients had high in-hospital mortality and short median survival time. Notably, we found that the mortality risk in the NRBC-positive group and the dynamic changes in NRBC count varied in subgroups with various underlying diseases.

The presence of NRBCs in peripheral blood was proven to indicate that there may be increased erythropoiesis, abnormal enucleation of nucleated erythrocytes, and hypoxic stress in vivo [10, 11]. Moreover, the production and/or release of NRBCs may be related to increased inflammatory cytokines [12]. In our research, we innovatively found that the existence of NRBC might also be due to low protein levels, hypercoagulability, and NRBC count positively correlated with alkaline phosphatase concentrations. Studies have demonstrated that low protein and systemic activation of blood coagulation are associated with inflammation and sepsis [13, 14]. In addition, alkaline phosphatase is an endogenous enzyme that plays a detoxification role by dephosphorylating bacterial endotoxins and proinflammatory mediators [15]. Studies have shown that alkaline phosphatase treatment in an animal sepsis model can reduce systemic inflammation and organ dysfunction and improve prognosis [16]. Therefore, we could infer that the leading causes of NRBC in ICU patients are inflammation and hypoxia.

A meta-analysis in 2008 showed that the total in-hospital mortality rate of ICU patients was 11–45% [17]. Moreno et al. reported that the mortality rate of ICU patients after discharge was between 5.0% and 25.0%, depending on the severity of the patients [18]. In a prospective study, the incidence of NRBC in ICU patients was 17.5% (67/383), and the mortality of NRBC-positive patients was 50.7%, which was significantly higher than that of NRBC-negative patients (9.8%) [19]. We also found that NRBC count was an independent risk factor for in-hospital mortality in ICU patients, which was consistent with the above studies.

In the subgroup analysis, we found that the NRBC-positive group had a higher mortality risk among patients without underlying diseases. This may be because the underlying conditions could cause NRBC positivity in peripheral blood because they are always combined with hypoxia and severe infection. Therefore, NRBCs in peripheral blood were primarily related to the presence or progression of the current illness in patients without the underlying conditions, which reduced the survival rate and survival time. In addition, for patients with cardiovascular and cerebrovascular diseases or digestive system diseases, it is necessary to more closely monitor whether peripheral blood NRBC is positive during the diagnosis and treatment process, which is conducive to a timely adjustment of the treatment plan and improvement of the prognosis.

The dynamic changes in NRBC showed a significant upward trend before death, and the tendency of patients in each subgroup was inconsistent. Studies have shown that hypertension and hypoxia can induce each other [20, 21]. Therefore, with the progression of the disease, patients with hypertension may be under severe hypoxia in various organs before death, which promotes the rapid generation of NRBCs and leads to a sharp increase in NRBCs in peripheral blood. Through case analysis, it can be speculated that if ICU patients have a high level of NRBC during hospitalization or show a rapid upward trend, it might indicate a poor prognosis.

There are some limitations to this study. First, the number of cases in this study was small. Second, because each patient's condition was different, it was impossible to perform the test every day, which led to the irregular time of NRBC detection and individual differences. Third, as the conditions of ICU patients were complex, they had different underlying diseases and their medication types and durations were also different, so the influence of medication use was not considered in the analysis process of this study. Therefore, more patients with proper medication use and subgrouping may be included to confirm the actual results.

## 5. Conclusion

The possible cause of NRBC in ICU patients was related to inflammation and hypoxia. The persistently high level and the sharp increase in NRBC count during hospitalization are predictors of in-hospital mortality in ICU patients, which can provide a new marker for the poor prognosis of ICU patients. The changing trend of the NRBC count varied in patients with different underlying diseases. NRBC dynamic monitoring is necessary for patients with cardiovascular and cerebrovascular diseases or digestive system diseases.

## Figures and Tables

**Table 1:** Comparison of clinical and laboratory data between NRBC-negative and NRBC-positive groups.

	NRBC-negative	NRBC-positive	*P* value
	131	46	

Sex (*n*, %)			
Male	92 (70.2)	27 (58.7)	0.152^b^
Female	39 (29.8)	19 (41.3)	

Age (years)	72.00 (58.50–82.00)	66.00 (55.00–78.75)	0.158

LOS (days)	13.00 (7.00–28.50)	10.00 (5.00–19.75)	0.067

Underlying diseases (*n*, %)			
Cancer	22 (16.8)	12 (26.1)	0.169^b^
Respiratory system disease	56 (42.7)	19 (41.3)	0.865^b^

Cardio-cerebrovascular disease	89 (67.9)	20 (43.5)	0.003^b^
Urinary system disease	24 (18.3)	16 (34.8)	0.022^b^
Digest system disease	30 (22.9)	13 (28.3)	0.466^b^
Hypertension	49 (37.4)	9 (19.6)	0.027^b^
Diabetes mellitus	27 (20.6)	8 (17.4)	0.791^b^

Clinical score			
APACHE II	19.62 ± 7.59	23.52 ± 9.39	0.005^a^
SOFA	8.00 (6.00–12.00)	13.50 (9.00–17.50)	<0.001
CCI	7.00 (5.00–8.50)	7.00 (6.00–8.00)	0.141

Medication (*n*, %)			
Blood transfusion	52 (39.7)	32 (69.6)	0.001^b^
Mechanical ventilation	113 (86.3)	37 (80.4)	0.480^b^
Hemodialysis/CRRT	13 (9.9)	11 (23.9)	0.033^b^
ECOMO	0 (0.0)	3 (6.5)	0.022^b^

Complete blood count			
WBC (×10^9^/L)	11.38 ± 5.23	12.34 ± 7.06	0.331^a^
N (×10^9^/L)	9.76 ± 5.04	10.42 ± 6.22	0.476^a^
RBC (×10^12^/L)	3.71 ± 0.79	3.25 ± 0.75	0.001^a^
Hb (g/L)	112.00 (93.50–128.50)	88.00 (78.44–105.88)	<0.001
RDW (%)	13.20 (12.62–14.09)	14.00 (13.41–15.85)	<0.001
NRBC (×10^9^/L)	0.00 (0.00–0.00)	0.52 (0.24–2.00)	<0.001
PLT (×10^9^/L)	192 ± 104	158 ± 108	0.059^a^

Coagulation function			
D-D (mg/L)	2.77 (1.44–7.49)	8.85 (2.71–15.38)	0.001
Fig (g/L)	4.19 ± 1.67	4.00 ± 1.71	0.506^a^

Blood gas			
PO_2_ (mmHg)	123 ± 38	113 ± 39	0.135^a^
PCO_2_ (mmHg)	33.87 (30.86–38.13)	33.38 (30.05–39.40)	0.880
SO_2_ (%)	98.30 (96.75–99.20)	97.10 (93.92–98.18)	<0.001
OI (mmHg)	316 ± 109	261 ± 111	0.004^a^

Liver function			
ALT (U/L)	20.47 (13.90–33.71)	64.15 (22.74–504.69)	<0.001
AST (U/L)	34.38 (21.14–68.10)	192.33 (42.14–626.17)	<0.001
ALP (U/L)	78.73 (63.03–106.63)	96.58 (75.50–173.39)	0.005
GGT (U/L)	31.70 (19.83–57.94)	56.87 (25.16–94.35)	0.007
TBIL (*μ*mol/L)	14.22 (9.20–19.46)	16.29 (10.06–43.00)	0.047
DBIL (*μ*mol/L)	5.11 (3.42–7.71)	7.82 (4.34–19.55)	0.003

Serum protein			
PA (mg/dL)	15.51 ± 7.82	10.55 ± 5.70	<0.001^a^
TP (g/L)	59.87 ± 10.87	53.83 ± 9.47	0.001^a^
Alb (g/L)	32.11 ± 6.35	27.28 ± 5.80	<0.001^a^
A/G	1.23 ± 0.35	1.07 ± 0.27	0.002^a^
TBA (*μ*mol/L)	2.41 (1.25–4.86)	5.26 (1.97–10.94)	<0.001
CHE (KU/L)	5.29 (3.69–6.70)	3.69 (2.04–5.25)	<0.001

Serum electrolyte			
K (mmol/L)	3.90 (3.74–4.34)	4.16 (3.75–4.73)	0.078
Na (mmol/L)	140.49 ± 5.81	142.62 ± 8.83	0.066^a^
Cl (mmol/L)	105.97 ± 6.38	106.17 ± 8.31	0.869^a^
Ca (mmol/L)	2.07 ± 0.16	2.00 ± 0.20	0.011^a^
Mg (mmol/L)	0.83 (0.74–0.91)	0.90 (0.78–1.00)	0.008
P (mmol/L)	1.00 (0.80–1.23)	1.16 (0.89–1.89)	0.035

Kidney function			
Crea (*μ*mol/L)	88.79 (57.57–143.94)	157.49 (83.39–281.06)	<0.001
BUN (mmol/L)	7.50 (5.18–13.04)	15.88 (8.60–20.91)	<0.001
UA (*μ*mol/L)	320.24 (224.18–426.63)	444.37 (323.05–562.86)	<0.001
eGFR (mL/min/1.73 m^2^)	71.00 (39.00–97.00)	34.50 (18.00–60.50)	<0.001

Lipid			
HDLC (mmol/L)	1.10 ± 0.37	0.83 ± 0.35	<0.001^a^
LDLC (mmol/L)	1.62 (1.12–2.23)	1.36 (0.87–1.96)	0.059
APOA1 (g/L)	1.06 ± 0.36	0.73 ± 0.29	<0.001^a^
APOB (g/L)	0.74 (0.56–0.95)	0.69 (0.50–0.88)	0.201
TC (mmol/L)	3.77 ± 1.98	3.06 ± 1.36	0.025^a^

Infection index			
CRP (mg/L)	61.04 (17.49–116.67)	107.34 (50.40–193.13)	0.003
SAA (mg/L)	247.20 (63.11–361.92)	284.63 (78.60–376.52)	0.615
PCT (ng/mL)	0.80 (0.18–5.42)	3.37 (1.01–15.53)	0.001

Myocardial enzyme			
LDH (U/L)	266.14 (207.22–392.32)	665.12 (402.18–1793.53)	<0.001

Complement			
C3 (g/L)	0.90 ± 0.26	0.72 ± 0.31	<0.001^a^

CRRT, continuous renal replacement therapy; ECOMO, extracorporeal membrane oxygenator; WBC, white blood cell; N, neutrophils; RBC, red blood cell; Hb, hemoglobin; RDW, red blood cell distribution width; NRBC, nucleated red blood cell; PLT, platelet; D-D, D-dimer; Fig, fibrinogen; PO_2_, partial pressure of oxygen; PCO_2_, partial pressure of carbon dioxide; SO_2_, oxygen saturation; OI, oxygenation index; ALT, alanine aminotransferase; AST, aspartate aminotransferase; ALP, alkaline phosphatase; GGT, *γ*-glutamine transferase; TBIL, total bilirubin; DBIL, direct bilirubin; PA, prealbumin; TP, total protein; Alb, albumin; A/G, albumin/globulin; TBA, total bile acid; CHE, cholinesterase; K, potassium; Na, sodium; Cl, chlorine; Ca, calcium; Mg, magnesium; P, phosphorus; Cr, crea; BUN, blood urea nitrogen; UA, uric acid; eGFR, estimated glomerular filtration rate; HDLC, high-density lipoprotein cholesterol; LDLC, low-density lipoprotein cholesterol; APOA1, apolipoprotein A1; APOB, apolipoprotein B; TC, total cholesterol; CRP, C-reactive protein; SAA, serum amyloid A; PCT, procalcitonin; LDH, lactate dehydrogenase; C3, complement 3. ^a^*P* value was obtained by the *t*-test; ^b^*P* value was obtained by the chi-square test; other *P* values were obtained by the Mann–Whitney U test.

## Data Availability

The datasets used during the current study are available from the corresponding authors upon request.

## Abbreviations

WBC: White blood cell
N: Neutrophils
RBC: Red blood cell
Hb: Hemoglobin
RDW: Red blood cell distribution width
NRBC: Nucleated red blood cell
PLT: Platelet
D-D: D-dimer
Fig: Fibrinogen
PO_2_: Partial pressure of oxygen
PCO_2_: Partial pressure of carbon dioxide
SO_2_: Oxygen saturation
OI: Oxygenation index
ALT: Alanine aminotransferase
AST: Aspartate aminotransferase
ALP: Alkaline phosphatase
GGT: *γ*-Glutamine transferase
TBIL: Total bilirubin
DBIL: Direct bilirubin
PA: Prealbumin
TP: Total protein
Alb: Albumin
A/G: Albumin/globulin
TBA: Total bile acid
CHE: Cholinesterase
K: Potassium
Na: Sodium
Cl: Chlorine
Ca: Calcium
Mg: Magnesium
P: Phosphorus
Cr: Crea
BUN: Blood urea nitrogen
UA: Uric acid
eGFR: Estimated glomerular filtration rate
HDLC: High-density lipoprotein cholesterol
LDLC: Low-density lipoprotein cholesterol
APOA1: Apolipoprotein A1
APOB: Apolipoprotein B
TC: Total cholesterol
CRP: C-reactive protein
SAA: Serum amyloid A
PCT: Procalcitonin
LDH: Lactate dehydrogenase
C3: Complement 3.
